# 2018 ISCB Innovator Award recognizes M. Madan Babu

**DOI:** 10.1371/journal.pcbi.1006164

**Published:** 2018-06-07

**Authors:** Christiana N. Fogg, Diane E. Kovats, Ron Shamir

**Affiliations:** 1 Freelance Science Writer, Kensington, Maryland, United States of America; 2 International Society for Computational Biology, Bethesda, Maryland, United States of America; 3 Blavatnik School of Computer Science, Tel Aviv University, Tel Aviv, Israel

**Figure:**
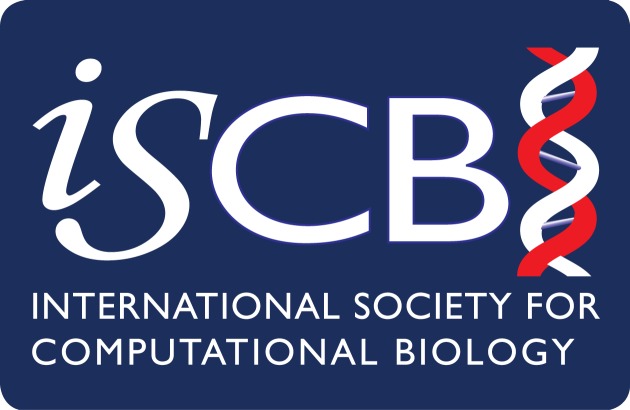


The International Society for Computational Biology (ISCB) Innovator Award recognizes an ISCB scientist who is within two decades of having completed his or her graduate degree and has consistently made outstanding contributions to the field of computational biology. The 2018 winner is Dr. M. Madan Babu, Program Leader at the Medical Research Council (MRC) Laboratory of Molecular Biology, Cambridge, United Kingdom. Madan will receive his award and deliver a keynote presentation at the 2018 International Conference on Intelligent Systems for Molecular Biology in Chicago, Illinois, being held on July 6–10, 2018.

## M. Madan Babu: Peering into the realm of regulation

**Fig 1:**
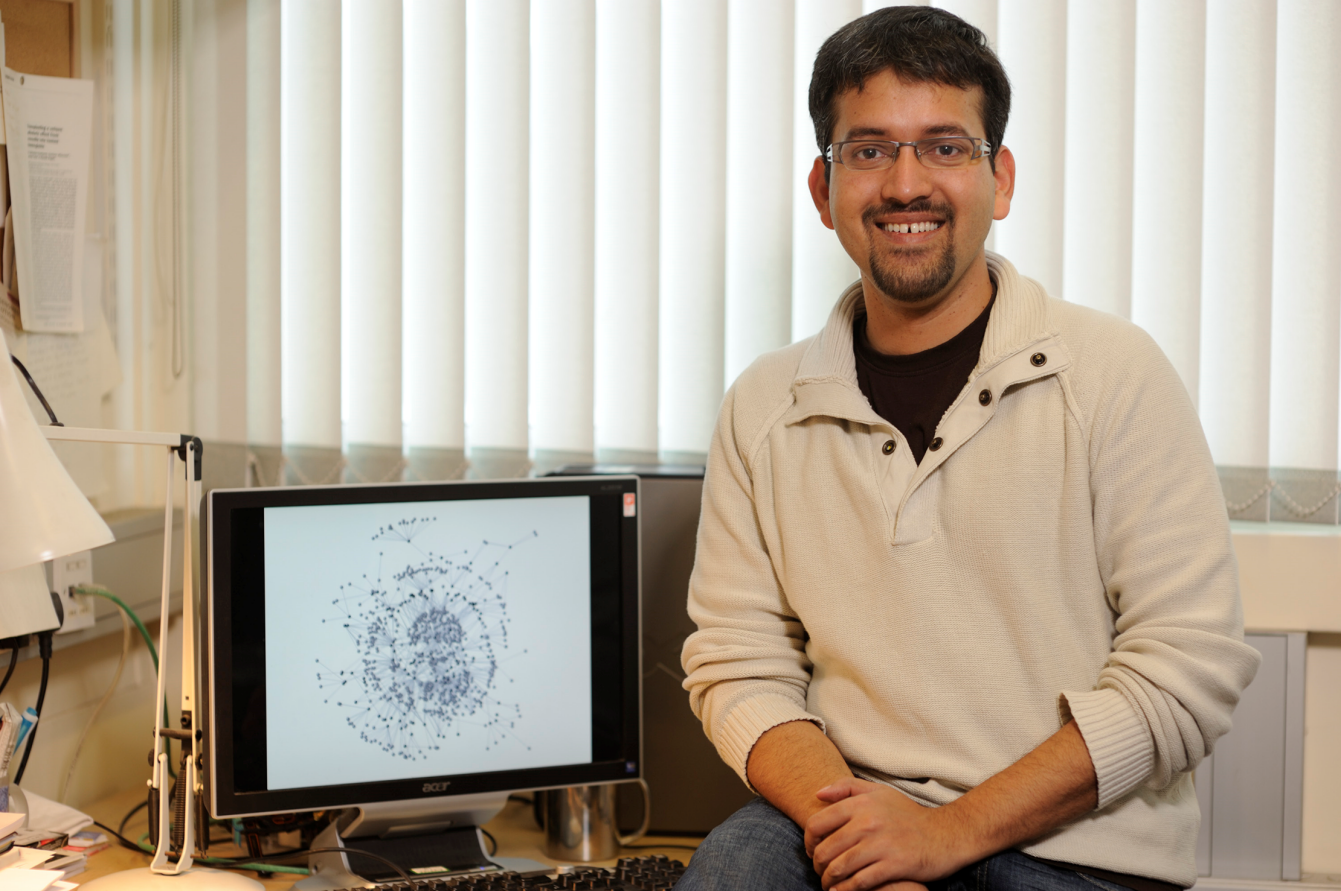
M. Madan Babu, MRC Laboratory of Molecular Biology. MRC, Medical Research Council.

M. Madan Babu (Fig 1) is the head of the Regulatory Genomics and Systems Biology group at the MRC Laboratory of Molecular Biology, Cambridge, UK. His work focuses on understanding how cellular systems are regulated at different scales (molecular, systems, and genomic levels) and how this impacts genome evolution.

Madan grew up in Chennai, India, and developed early interests in computer science and biotechnology. As a young child, he has vivid memories of his father bringing home a personal computer, and soon after, he became interested in learning to program. He also remembers when his family first started using the internet, and recalled, “In the mid-90s, we started having access to the internet. This made a big difference in the days where access to information beyond textbooks was not readily available; so, thanks to my father, I had these opportunities early in my life.” Madan discovered biotechnology as a high school student and attributes his lifelong interest in biology to the impact of his biology teacher, Dr. M. C. Aruna, who discussed foundational biological concepts with him, including how genetic information can be used to understand living systems.

Madan went on to pursue a Bachelor of Technology (Biotechnology) degree at Anna University, Center for Biotechnology in Chennai, India. He first became aware of computational biology during a year-long undergraduate research internship, at which time he was exposed to the work of Cyrus Chothia and Arthur Lesk in a course on protein structure. He became fascinated with this research area and then delved into seminal papers on computational genomics, protein engineering, and structural bioinformatics. As an intern, Madan pursued undergraduate research under the guidance of Professor Balaram and Professor K. Sankran and saw this as a key turning point in his career path. He recollected, “We started applying methods from computer science to study protein sequences and structures. For the first time, I experienced how to define a scientific problem, develop computational methods to solve it, and write up and defend the findings for publication. This really got me excited, and that was when I decided that I would like to pursue a career in computational biology”.

Madan recognizes that his interest in computational biology was fostered by his ability to access publicly available protein and genomic data on his own computer, as well as the open access he had to lecture materials, methods, and algorithms from computational biologists spanning the globe. He said, “I cannot forget the day when I wrote an email to Research Collaboratory for Structural Bioinformatics (RCSB) from India and received a 5-part CD-ROM with coordinate data for all protein structures. Being able to look at protein structures using RASMOL from home and writing FORTRAN programs to analyze structures as an undergraduate student was one of the most exciting experiences that really captured my interest in the field”.

Madan left India in 2001 to pursue his PhD in computational genomics at the MRC Laboratory of Molecular Biology and Trinity College, University of Cambridge, UK, under the guidance of Dr. Sarah Teichmann. His PhD research explored various aspects of gene regulatory networks and marked the beginning of a very fruitful mentorship under Teichmann. Madan carried out his postdoctoral training at the National Center for Biotechnology Information, National Institutes of Health (NIH) in Bethesda, Maryland, United States of America, under the guidance of Dr. L. Aravind, during which time he learned the importance of having broad interests in diverse subject areas as well as critically analyzing the complexity of biological systems at every possible level of detail.

After a brief but extremely productive postdoctoral fellowship, Madan became a group leader at the age of 26 of the Regulatory Genomics and Systems Biology Group at the MRC Laboratory of Molecular Biology in 2006. As a principle investigator (PI), he has come to appreciate how his team of scientists can work together to tackle scientific questions on a much larger scale and shed new light on long-standing, fundamental questions. He said, “One of the things that I really enjoy about the field of computational biology is that you really integrate knowledge from various disciplines—biology, statistics, computer science, mathematics, physics, and chemistry. This means our lab is an amalgamation of people across disciplines that are really passionate about using interdisciplinary approaches to solve the problems they are working on”.

Madan’s group currently focuses on several areas of research, including studies on G-protein–coupled receptors (GPCRs), a protein family involved in almost every aspect of human physiology and targeted by numerous drugs. Madan’s group is also using a combination of computational and experimental approaches to discover which parts of unstructured protein regions are functional and understand what makes them functional. His group is interested in applying developments in statistical learning and advances in large-scale genome sequencing to better understand natural variation in the human population as well as gain insight into how genomic variation impacts rare and common diseases.

Madan is greatly honored to be selected as the recipient of the 2018 ISCB Innovator Award. He is grateful for his academic mentors and colleagues, including Sarah Teichmann, L. Avarind, Cyrus Chothia, Michael Levitt, Veronica Van Heyningen, Eugene Koonin, Stephen Michnick, Richard Kriwacki, Uri Alon, Gebhard Schertler, Peter Wright, Keith Dunker, Janet Thornton, Tom Blundell, and Venki Ramakrishnan, who have inspired him through their work and/or provided him valuable advice at various stages of his career. He is also appreciative of his past and present group members and the MRC Laboratory of Molecular Biology for the freedom to develop new skills and take risks in pursuing research that pushes scientific boundaries.

